# Exploring Natural Variations in *Arabidopsis thaliana*: Plant Adaptability to Salt Stress

**DOI:** 10.3390/plants13081069

**Published:** 2024-04-10

**Authors:** Marco Lombardi, Manuel Bellucci, Sara Cimini, Vittoria Locato, Francesco Loreto, Laura De Gara

**Affiliations:** 1 Unit of Food Science and Nutrition, Department of Science and Technology for Sustainable Development and One Health, Università Campus Bio-Medico di Roma, Via Alvaro del Portillo 21, 00128 Rome, Italy; marco.lombardi@agro.au.dk (M.L.); m.bellucci@unicampus.it (M.B.); s.cimini@unicampus.it (S.C.); l.degara@unicampus.it (L.D.G.); 2 Department of Biology, Agriculture, and Food Sciences, National Research Council of Italy (CNR-DISBA), Piazzale Aldo Moro 7, 00185 Rome, Italy; 3 National Biodiversity Future Center, NBFC, 90133 Palermo, Italy; francesco.loreto@unina.it; 4 Department of Biology, University of Naples Federico II, 80138 Naples, Italy; 5 Institute for Sustainable Plant Protection, National Research Council of Italy (CNR-IPSP), Via Madonna del Piano 10, 50019 Sesto Fiorentino, Italy

**Keywords:** salinity, root system architecture, reactive oxygen species, *Arabidopsis thaliana*, biodiversity

## Abstract

The increase in soil salinization represents a current challenge for plant productivity, as most plants, including crops, are mainly salt-sensitive species. The identification of molecular traits underpinning salt tolerance represents a primary goal for breeding programs. In this scenario, the study of intraspecific variability represents a valid tool for investigating natural genetic resources evolved by plants in different environmental conditions. As a model system, *Arabidopsis thaliana,* including over 750 natural accessions, represents a species extensively studied at phenotypic, metabolic, and genomic levels under different environmental conditions. Two haplogroups showing opposite root architecture (shallow or deep roots) in response to auxin flux perturbation were identified and associated with EXO70A3 locus variations. Here, we studied the influence of these genetic backgrounds on plant salt tolerance. Eight accessions belonging to the two haplogroups were tested for salt sensitivity by exposing them to moderate (75 mM NaCl) or severe (150 mM NaCl) salt stress. Salt-tolerant accessions were found in both haplogroups, and all of them showed efficient ROS-scavenging ability. Even if an exclusive relation between salt tolerance and haplogroup membership was not observed, the modulation of root system architecture might also contribute to salt tolerance.

## 1. Introduction

In recent decades, the world has been experiencing a strong change in climatic conditions mainly caused by anthropogenic factors. One of the main features characterizing climate change is represented by global warming and the concomitant change in precipitation patterns (https://www.ipcc.ch/report/sixth-assessment-report-cycle/accessed on 19 March 2023). In particular, rising sea levels, reduced rainfalls, and increasing evapotranspiration are causing a sharp increase in soil salinization, affecting many agricultural areas [1]. In view of the expected increase in the world population by 2050, food demand is predicted to increase by approximately 80% [2], calling for the intensification of production under growingly adverse environmental conditions.

The harmful impact of salinity on plant productivity requires identifying traits conferring tolerance and improving resilience. Identifying and exploiting these traits can pave the way for developing salt-tolerant plant varieties in the face of challenging environmental conditions [3]. In this context, natural accessions, which survive under evolving climate change, represent a reservoir of genes involved in adaptive mechanisms [4].

Roots are directly exposed to salt stress, and they are the primary plant organ able to perceive the deleterious accumulation of salts in the soil and rapidly respond to salt stress by sending signals throughout the plants and shifting nutrients [5]. Root system architecture (RSA) describes the spatial organization of roots in the soil and thus primarily influences a plant’s ability to capture mobile and immobile resources, which sets the plant’s growth performance [6]. In *A. thaliana*, salinity affects RSA traits such as primary root (PR) length, lateral root (LR) formation, and root orientation [5].

Recently, 215 *Arabidopsis thaliana* natural accessions were classified into two different genetic clusters, namely haplogroups, based on their response to the auxin transport inhibitor *N*-1-naphtylphthalamic acid (NPA) in terms of root growth direction [7]. In particular, 199 accessions showed a common (C) response to NPA (horizontal root growth), and 16 accessions showed a high (H) response to NPA (vertical root growth) [7]. These different RSA configurations were related to the allelic variations revealed by genome-wide association analysis in the EXO70A3 locus. The accessions belonging to the different haplogroups exhibit varying levels of fitness in environments with fluctuating rainfall patterns. However, the impact of salt stress on the two identified haplogroups is unclear.

Additionally, salinity stress often leads to an increase in reactive oxygen species (ROS) production in plant cells [8]. ROS, including molecules like superoxide radicals and hydrogen peroxide, are generally harmful, but they can also serve as signals in stress responses [9]. Natural populations may possess traits that enhance plant ability to counteract ROS build-up or mitigate the harmful effects of ROS under saline conditions [10]. These defensive mechanisms involve increased expression of antioxidant systems playing a key role in neutralizing ROS and maintaining cell redox homeostasis [11].

Here, an intraspecific phenotype screening on eight accessions of *A. thaliana* belonging to both previously identified haplogroups was performed in control conditions and under moderate (75 mM NaCl) and severe (150 mM NaCl) salt stress. The behavior of four *A. thaliana* natural accessions belonging to the two haplogroups and showing different salt susceptibility (Col-0, Sha, Ty-0, MIB-60) was investigated in depth in order to highlight potential effects of EXO70A3 locus variations on RSA modulation under salt stress. The selected accessions showed different accumulations of ROS in roots and gene expression modulation of selected redox-related genes under both control and moderate salt stress conditions.

## 2. Results and Discussion

### 2.1. Screening of Natural Arabidopsis thaliana Accessions for Salt Sensitivity

In order to study intra-species variability in terms of salt stress susceptibility, eight natural accessions of the model plant *A. thaliana* were used. Previously, the same natural accessions were classified according to their response to the auxin transport inhibitor *N*-1-naphtylphthalamic acid (NPA) [7]. Four genotypes selected for this study (Col-0, Ler-1, Lp2-2, Sha) belong to haplogroup C, showing agravitropic root growth (shallow roots) in response to NPA treatment, whereas four genotypes (Coc-1, LAC-5, MIB-60, Ty-0) belong to the haplogroup H showing deeper roots following NPA treatment, compared to roots grown under control conditions [7]. These accessions were subjected to NaCl exposure at their seedling stage, and the effect of salt on primary root growth was investigated over 5 days of treatment.

**Figure 1:**
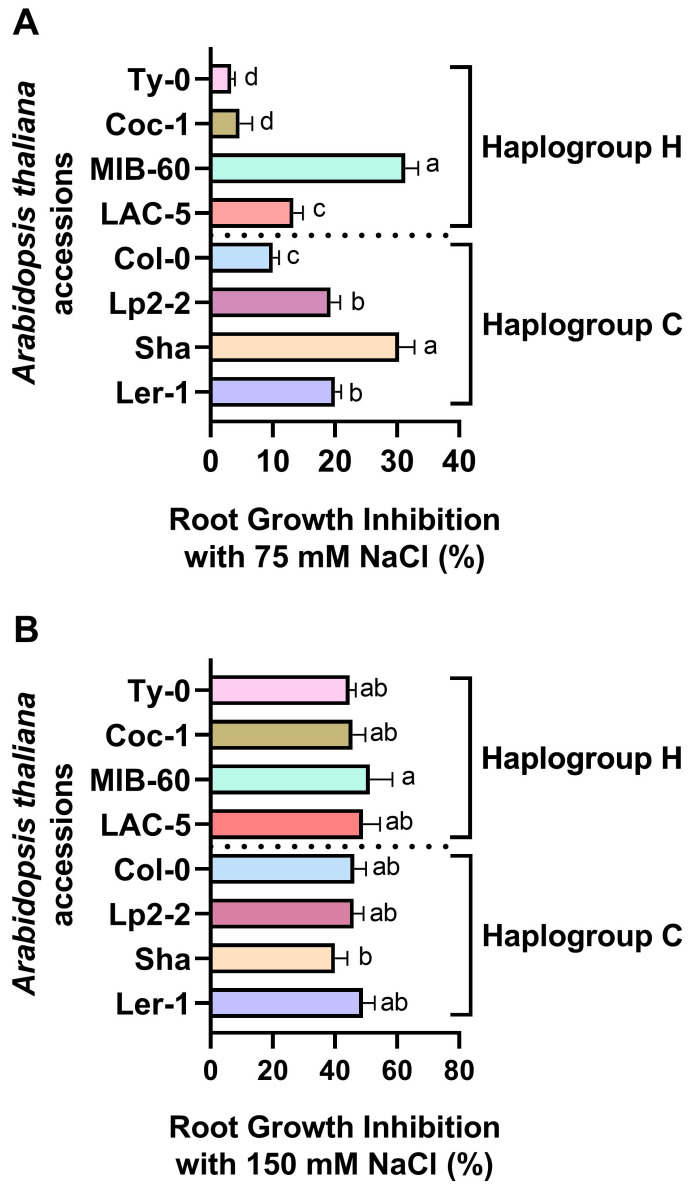
Primary root growth inhibition in *Arabidopsis thaliana* accessions exposed to 75 mM (**A**) and 150 mM (**B**) NaCl. The percentage of root growth inhibition was calculated by comparing the increase in primary root length of plants exposed to 5 days of salt treatment to the primary root length increase observed at the same time in plants grown under control conditions. Different letters indicate statistically different values among accessions according to ANOVA (*p* < 0.01).

Exposure of accessions to severe salt stress (150 mM NaCl) caused a drastic reduction in primary root length in all treated plants. Root growth inhibition ranged from 40 to 51% compared to the growth of primary roots of control plants without showing significant differences among lines (*p* > 0.05; Figure 1B). Therefore, 150 mM NaCl treatment did not allow for the distinction of different phenotypes in response to salt stress. On the other hand, exposure to moderate salt stress (75 mM NaCl) differently affected primary root growth depending on the tested accession. As shown in Figure 1A, both haplogroups presented accessions showing contrasting salt sensitivity. Further analyses were carried out on accessions belonging to the two haplogroups showing opposite sensitivity to salt stress in terms of primary root growth inhibition. Col-0 (member of haplogroup C) and Ty-0 (member of haplogroup H) were selected as tolerant lines, retaining, respectively, 90 ± 2 and 95 ±1.8% of primary root growth when treated with 75 mM NaCl, compared to control conditions (Figure 1), whereas Sha (haplogroup C) and MIB-60 (haplogroup H) were selected as sensitive accessions, showing primary root growth inhibition when treated with 75 mM NaCl, respectively of 30.3 ± 2 and 31.3 ± 3%, compared to controls (Figure 1). Moreover, primary root growth inhibition was observed already after one day of salt treatment only in the sensitive accessions Sha (36 ± 4%) and MIB-60 (27 ± 3%), compared to control conditions (*p* < 0.01).

Depending on stress intensity, plants can experience aboveground and belowground growth inhibition to different extents. This mainly depends on reduced carbon acquisition via photosynthesis, as well as on the reallocation of the resources toward defense responses instead of growth [12]. In particular, soil salinization reduces the capability of plants to absorb water, reduces stomatal conductance, and, consequently, reduces CO_2_ availability for photosynthesis and growth [13]. Consequently, reducing equivalents generated using photochemical reactions and not used by photosynthesis trigger over-reduction in photosystems, promoting ROS accumulation and cell component damage. Moreover, Na^+^ competition with K^+^ for root uptake disrupts cell homeostasis, altering turgor maintenance and membrane potential, as well as enzyme activity [14]. Zhang et al. [12] proposed that growth inhibition in *A. thaliana* plants under stress, including salt stress, also depends on reduced mitotic activity, which occurs as a consequence of the downregulation of cyclins’ transcripts and concomitant up-regulation of the expression of genes coding for cyclins’ inhibitors [15]. Therefore, root growth of salt-stressed plants seems to be inhibited by cell cycle arrest, which reduces the number of elongating cells and, consequently, root size [15]. Taking into account these considerations, root growth inhibition appears to be associated with plant stress perception, and it can be a useful parameter to classify genotypes in terms of their salt stress sensitivity.

### 2.2. Effect of Salt Stress on Arabidopsis thaliana Root Architecture

**Figure 2:**
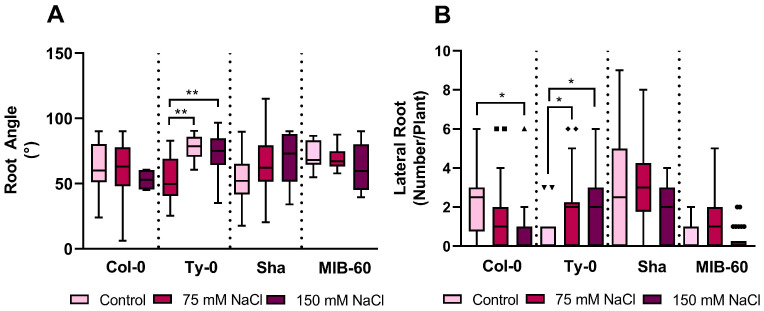
Root architecture traits in *Arabidopsis thaliana* accessions belong to haplogroups C and H and show contrasting sensitivities to salt. Measurements on tolerant (Col-0, Ty-0) and salt-sensitive accessions (Sha and MIB-60) were carried out in 5-day-old seedlings maintained under control conditions or after 5 days of moderate (75 mM NaCl) and severe (150 mM NaCl) salt treatment. (**A**) Root angle relative to soil surface simulated using nylon mesh (see Material and Methods for details). (**B**) Number of newly developed lateral roots per plant. * *p* < 0.05 ** *p* < 0.01 according to the *t* student test.

Root architecture is crucial in determining plant tolerance to drought, osmotic, and salt stresses [3,16]. Root angle and lateral roots’ density have been selected as the main traits defining root architecture in plants [17]. We investigated whether these two traits were affected by salt stress in the four accessions belonging to the two haplogroups and with contrasting sensitivity to salt in terms of primary root growth inhibition. Both parameters significantly increased only in the salt-tolerant Ty-0 line as a consequence of NaCl treatments (Figure 2A,B).

The increase in root angle is compatible with the capability of this *A. thaliana* accession of haplogroup H to orientate roots in deep soil when auxin flux is perturbed by NPA [7]. Deeper roots can potentially explore soil better than shallow ones [16], making this trait a desirable feature in plants coping with water and nutrient deficits [18].

The two salt-tolerant lines of different haplogroups (Col-0 and Ty-0) reached similar primary root lengths after five days of salt treatment (2.3 ± 0.5 and 2.2 ± 0.4 cm, respectively). However, the number of lateral roots decreased in Col-0 plants under NaCl severe stress, whereas Ty-0 showed a higher number of lateral roots normalized per plant under both salt conditions compared to the control situation (Figure 2B). In Ty-0 accession, the increased density of lateral roots may influence the plant’s capability to cope with salt by expanding the root surface available for water and nutrient absorption.

Root branching has been related to auxin flux changes under abiotic stress [19]. Salt stress reduces the expression of genes coding for the root auxin transporters (the PIN family proteins, PIN 1 and PIN 2) at the root tip level, suggesting that auxin flux is perturbed in response to salt [20]. The accessions analyzed in this study were clustered depending on the allelic variation of the EXO70A3 locus, an exocytosis factor acting on PIN 4 distribution. It has been suggested that EXO70A3 locus variants can have an adaptive role in specific environmental conditions, particularly under drought stress. In particular, Ogura and co-workers [7] hypothesized that the capability to form shallow roots by haplogroup C accessions represents an adaptive mechanism working in the short term under water deficit. This mechanism would make plants growing in ecosystems characterized by sparse rainfalls able to capture water when it is available, whereas root gravitropic growth in haplogroup H accessions may improve tolerance to drought stress in the long term.

Our results, showing increased lateral root formation only in the tolerant accession of haplogroup H (Ty-0) under salt stress, suggest that PIN distribution can be very flexible, possibly involving other unknown regulative factors.

### 2.3. Reactive Oxygen Species’ Profile and Modulation in Salt-Stressed Arabidopsis thaliana Roots

**Figure 3:**
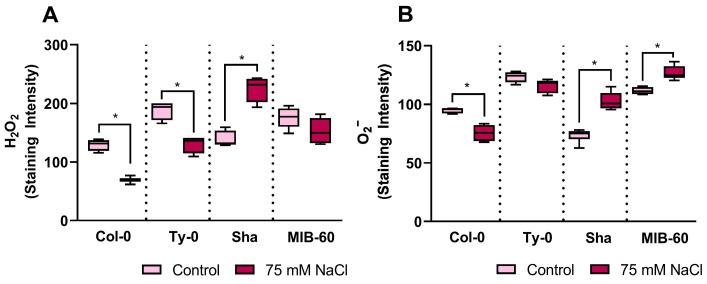
ROS accumulation in the roots of *Arabidopsis thaliana* accessions 30′ after 75 mM NaCl exposure (**A**) Superoxide anion level was determined as NBT staining intensity in root tips under control and treated conditions (75 mM NaCl). (**B**) Hydrogen peroxide level was determined as BES-H2O2-Ac staining intensity in root tips under control and treated conditions (75 mM NaCl). * *p* < 0.01 according to the *t* student test.

Exposure of plants to salt stress promptly causes ROS hyper-production before phenotypic evidence occurs [21,22,23]. Consistently, the primary roots of the salt-sensitive accessions of both haplogroups experienced a rapid ROS increase when subjected to 75 mM NaCl treatment, which was estimated as the most appropriate tested salt stress condition to distinguish tolerant and sensitive accessions (Figure 3). In particular, both salt-sensitive accessions (Sha and MIB-60) experienced an increase in superoxide anion levels in primary roots subjected to 75 mM NaCl treatment (Figure 3B), and Sha also presented higher hydrogen peroxide levels in roots under salt compared to control conditions (Figure 3A). On the other hand, tolerant accessions (Col-0 and Ty-0) showed a reduction in the level of hydrogen peroxide in primary root under salt treatment (Figure 3A), and Col-0 also presented a decrease in superoxide anion in roots subjected to salt treatment (Figure 3B).

ROS plays a crucial role in plant development as well as in plant response to stress [11,24,25,26]. In *Arabidopsis thaliana*, root development seems to involve a pattern of ROS distribution. Superoxide anion mainly accumulates in the meristematic zone, whereas hydrogen peroxide mainly accumulates in the elongation zone. A change in the ratio of superoxide and hydrogen peroxide sets the end of cell division in the transition zone and the start of cell differentiation. Indeed, a reduced meristem size can be observed in plants where the ROS pattern was manipulated by treatments with superoxide anion inhibitors or by genetic approaches [27].

A gradient of redox potential has also been described at the root tip level. Salt stress, which generally reduces the proliferation activity of meristematic cells, also reduces the root redox gradient, making the whole root tip more oxidized [20]. Simultaneously, salt treatments reduce PIN genes’ expression and PIN 1 and PIN 2 membrane allocation at the root tip level, also suggesting a concomitant perturbation in auxin flux and redox patterns under salt stress [20,28].

**Figure 4:**
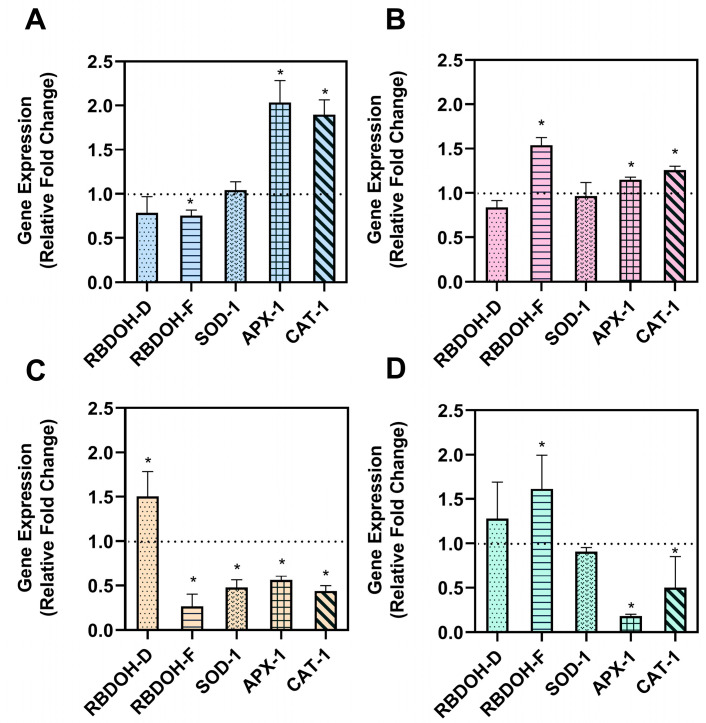
Transcript level of redox genes in the roots of *Arabidopsis thaliana* accessions of haplogroups C and H 30′ after 75 mM NaCl exposure. Transcript level of Respiratory burst oxidase homolog D (RBDOH-D), Respiratory burst oxidase homolog F (RBDOH-F), Superoxide Anion Dismutase 1 (SOD-1), Ascorbate Peroxidase 1 (APX-1) and Catalase 1 (CAT-1) genes are shown. Panels refer to salt-tolerant accessions Col-0 (**A**) and Ty-0 (**B**) and to salt-sensitive accessions Sha (**C**) and MIB-60 (**D**). The value of each transcript level was normalized for the transcript level of actin 7 as a reference gene and expressed as a fold change of salt-stressed roots compared to the relative control. * *p* < 0.05 according to the *t* student test.

Plant capability to control ROS levels mainly depends on the balance between the induction of ROS scavenger enzymes and the activity of ROS-producing enzymes. The upregulation of antioxidant systems has often been reported as part of plant defense responses activated toward environmental stress, triggering ROS over-production [10]. Consistently with the general decrease in ROS levels in the salt-tolerant accessions, the genes coding for hydrogen peroxide scavenger enzymes CAT1 and APX1 were highly expressed, whereas the transcript of RBOH-F, the enzyme involved in superoxide anion production, was down-regulated in Col-0 roots subjected to salt stress (Figure 4A). A moderate salt-dependent up-regulation of the expression of the genes coding for ROS scavenger enzymes was also recorded in Ty-0 roots (Figure 3B). The RBOH-F gene was also up-regulated in salt-stressed Ty-0 roots (Figure 4B), which may partly explain why the superoxide anion did not decrease in this case (Figure 3B).

In the salt-sensitive accessions, the expression of the investigated redox genes also matched the increase in ROS level in NaCl-stressed primary roots (Figure 3A,B). In particular, RBOH-D transcript was up-regulated in Sha, and the transcripts of the ROS scavenger enzymes SOD 1, APX1 and CAT1 were down-regulated in the same accession under salt stress (Figure 4C), whereas in MIB-60 salt-stressed primary roots, RBOH-F transcript was also up-regulated, explaining superoxide anion accumulation, and APX 1 and CAT 1 gene expression decreased, in line with the sustained level of hydrogen peroxide (Figure 3 and Figure 4D).

The production of ROS under salt stress has been previously linked to AtRBOHD expression [29]. This enzyme catalyzes the synthesis of superoxide anion that can be converted to hydrogen peroxide by superoxide dismutases (SOD). By crossing membranes, moderate levels of hydrogen peroxide can work as a mobile signal to trigger defense responses [30]. The transcription factor ABI4 activates the expression of AtRBOHD under salt stress, and both *abi4* and *rbohd* mutants are salt-insensitive, whereas the correspondent over-expressing lines are hypersensitive to salt [31]. This evidence suggests that salt tolerance is dependent on reduced ROS accumulation, as also observed in our experiments. However, the double mutant *atrbohd atrbohf* is more sensitive to salt than the wild type [32], probably reflecting ROS activity as key signal molecules when they are present at low concentrations [33,34]. Indeed, ROS timing and intensity appear to be crucial features for tuning the role of these compounds in response to salt stress [35,36].

## 3. Materials and Methods

### 3.1. Plant Materials, Growth Conditions, and Root Phenotyping

Eight natural accessions of *A. thaliana* previously classified in two haplogroups because of their different response to the auxin transport inhibitor *N*-1-naphtylphthalamic acid (NPA) were selected for this study [7]. Based on this classification, four genotypes belong to the haplogroup C since they showed a common response to NPA (Col-0, Ler-1, Lp2-2, Sha), whereas four genotypes belong to the haplogroup H showed high response to NPA (Coc-1, LAC-5, MIB-60, Ty-0). All these accessions were purchased from Nottingham Arabidopsis Stock Center—NASC (Leicestershire, UK), except for Coc-1, which was a gift from SALK Institute (San Diego, CA, USA). Seeds of *Arabidopsis thaliana* were surface sterilized with 70% ethanol and 15% bleach solution (NaClO 14%, Triton X-100 0.2%) for 2 and 10 min, respectively, and then washed several times with sterile water. Seeds were stratified at 4 °C for 3 days [37].

All plants were grown on nylon mesh (1000 μm opening; 1 cm × 11.5 cm) [38] on a medium containing ½ Murashige and Skoog salt mixture, 0.5% sucrose, 0.8% (*w*/*v*) agar, 5.7 pH (solid MS medium, 40 mL/plate) in squared sterile Petri plates that were sealed with sterile Biofilm tape. The obtained plates were placed vertically in a growth room at 23 °C, 60% humidity, with a 16/8 h light/dark photoperiod with fluorescent light (100 μmol m^−2^ s^−1^) [39]. 5-day-old seedlings were then transferred with nylon mesh to plates containing fresh solid MS medium (40 mL/plate) supplemented with 75 mM (moderate salt stress) and 150 mM (severe salt stress) NaCl in the top segment of squared sterile Petri plates that were sealed with sterile Biofilm tape and grown vertically for 5 days after treatment (5 DAT). For control conditions, 5-day-old seedlings were transferred with nylon mesh in new plates containing solid MS medium (40 mL/plate) in the top segment of squared sterile Petri plates that were sealed with sterile Biofilm tape and grown vertically for 5 DAT. Over the plant growing period, the roots were kept in the darkness and the shoot in the light, as in the D-root system described by [40]. To shade the roots, an aluminum holder was adapted to the shape of the plate, covering up to 2 cm from the top of the plate. All experiments were repeated three times.

Images of plants were acquired after NaCl treatments and under control conditions using an EPSON Perfection V850 pro flat scanner [41]. For each genotype and experimental condition, 15 plants were used, and the whole experiment was repeated three times. Primary root length (PR), root angle (RA), and lateral root number normalized for plant (LR/plant) were analyzed using the Fiji software package of ImageJ version 1.54f, 29 June 2023 [42].

### 3.2. ROS Assays

For hydrogen peroxide detection, 5-day-old seedlings of Col-0, Ty-0, MIB-60, and Sha accessions grown on solid MS medium were transferred in liquid MS medium (control) or liquid MS medium supplemented with 75 mM NaCl for 30 min (moderate salt stress). Then, the roots were incubated in a liquid MS medium containing 50 mM of BES-H_2_O_2_-Ac (WAKO) for 30 min in the dark [27]. Roots were observed using a Nikon laser scanning confocal microscope setting excitation wavelength at 485 nm and detection wavelength at 515 nm. Maximum projection images were generated for BES-H_2_O_2_-Ac stained roots. Confocal images were processed, stitched, and analyzed using the Fiji package of ImageJ version 1.54f, 29 June 2023. The average intensity of BES-H_2_O_2_-Ac was measured in about 18 biological replicates for each analyzed accession [43].

For superoxide anion detection, 5-day-old seedlings of Col-0, Ty-0, MIB-60, and Sha accessions were transferred in liquid MS medium for control and moderate salt stress treatments as described for hydrogen peroxide detection. Roots were then stained for 2 min in 20 mM phosphate buffer (pH 6.1) containing 200 μM NBT in the dark and rinsed twice with distilled water [44]. Images for NBT staining were obtained using a ×10 objective of a Nikon light microscope. The total intensities of NBT staining in the root tip were measured in 18 biological replicates for each analyzed accession using the Fiji software package.

### 3.3. RNA Extraction and Gene Expression Analysis

Five-day-old roots grown on solid MS medium were soaked with liquid MS medium in the absence (control) or in the presence of 75 mM NaCl (moderate salt stress) for 30 min. Root tips (1 g for each accession and treatment) were then harvested. The experiment was performed three times on different biological replicates. Total RNAs were extracted using TRIzol reagent (Ambion, Austin, TX, USA), and the first strand cDNAs were synthesized using a Reverse Transcriptase kit according to the provided protocols. qRT-PCR was performed using the Thermofisher Real-Time System (Thermofisher Scientific, Waltham, MA, USA) using SYBR Green mix. qRT-PCR was used to detect the gene expression in the 5-day-old root tips of ROS-related genes. Actin 7 was used as a reference gene. The PCR amplification was performed with the procedure of 1 min at 95 °C, 50 cycles of 10 s at 95 °C, 15 s at 55–60 °C and 15 s at 72 °C, 5 min at 72 °C [43,45]. Primers’ sequences are reported in Table S1.

### 3.4. Statistical Analysis

Differences among treatments or accessions were assessed using Student’s *t*-test or two-way ANOVA with post hoc Tukey’s test. The values were considered statistically significant, at least for *p* < 0.05. All the measurements were performed at least three times.

## 4. Conclusions

Intraspecific variability has been investigated to possibly increase the knowledge of the mechanisms involved in plant response to salt stress with reference to root growth and architecture. Salt-tolerant accessions were found in both haplogroups characterized by opposite root growth directions in response to chemically induced auxin flux perturbation (horizontal or gravitropic). However, only the salt-tolerant accession Ty-0, belonging to haplogroup H, characterized by the gravitropic response, went deeper underground in response to salt compared to control conditions.

Salt-sensitive accessions of both haplogroups showed higher ROS accumulation than tolerant ones and down-regulation of genes coding for ROS scavenger enzymes, while the opposite was observed in salt-tolerant genotypes. ROS accumulation is therefore here confirmed as an important determinant of salt susceptibility in the investigated *A. thaliana* accessions. However, the different trends of hydrogen peroxide and superoxide anion observed among the accessions belonging to C and H haplogroups underline the complex role of ROS signature in balancing stress response and growth regulation.

Ty-0 showed the lowest susceptibility to salt stress in terms of primary root growth inhibition, and it was the most responsive accession to salt stress in terms of RSA modulation via expression of genes controlling ROS production and scavenging, suggesting a fine-tuned modulation of the redox system in this genotype.

Since ROS accumulation under salt stress primarily depends on osmotic unbalance and Na^+^ toxicity, further analyses are required to assess if Ty-0’s success in keeping low ROS level also depends on its capability to avoid Na^+^ accumulation and/or to preserve root water content activating mechanisms involved in osmotic regulation.

## Data Availability

The data presented in this study are available on request from the corresponding author.

## Supplementary Materials

The following supporting information can be downloaded at: https://www.mdpi.com/article/10.3390/plants13081069/s1, Table S1: Primers’ list.
