# Supplementary material for: Exploring Natural Variations in Arabidopsis thaliana: Plant Adaptability to Salt Stress
Source: Plants (Basel). 2024 Apr 10;13(8):1069. doi: 10.3390/plants13081069 (PMC11054533; doi:10.3390/plants13081069)
Supplement: Supplementary file 1 [file plants-13-01069-s001.zip › plants-2940505-supplementary.pdf]

Table S1. Primers' list.

Gene	Gene Reference	Forward primer	Reverse primer	Ref.
RBOHD	AT5G47910	GTACACCCACCATTTGTTCATC	AAAGCACGGAGCAGCCT	[43]
RBOHF	AT1G64060	GACACGCCAAGACGAAAGA	ACACCCCGTTGGTCAAGTT	[43]
SOD1	AT3G10920	GAACCTTGCTCCTTCCAGTG	TCTTCAGTTCTTTGTCTAGTCCG	[43]
CAT1	AT1G20630	GCCCCTAAATGTGCTCACC	AAGCACTTCTCACGATTTCCA	[43]
APX1	ATLG07890	AGAAGGCTGTTGAGAAGTG	CAGGGTGGAAAGGAATGT	[45]
ACTIN7	AT5G09810	GGCCGATGGTGAGGATATTCAGCCACTTG	TCGATGGACCTGACTCATCGTACTCACTC	[43]
